# Multi-omics analysis identifies gut microbiota-glutamine axis contributing to the pathogenesis of reflux esophagitis

**DOI:** 10.3389/fmicb.2026.1805181

**Published:** 2026-05-25

**Authors:** Yu Wu, Qi Gao, Huichao Yang, Yu Wang, Lin Lang, Bin Liu, Xiaolin Jiang, Di Li, Ximo Wang, Jing Xun, Qi Zhang

**Affiliations:** 1 Tianjin NanKai Hospital, Tianjin Medical University, Tianjin, China; 2 Tianjin Key Laboratory of Acute Abdomen Disease Associated Organ Injury and ITCWM Repair, Tianjin, China; 3 Institute of Integrative Medicine for Acute Abdominal Diseases, Tianjin, China

**Keywords:** glutamine, glutamine-glutamate metabolic pathway, gut microbiota, inflammatory mechanism, reflux esophagitis

## Abstract

**Background:**

Reflux esophagitis (RE), a common gastroesophageal reflux disease characterized by esophageal mucosal inflammation, is closely associated with gut microbiota dysbiosis and metabolic abnormalities. The glutamine-glutamate metabolic pathway regulates inflammation and mucosal barrier function, but its role in RE and association with gut microbiota remain unclear. This study aimed to characterize gut microbiota and serum metabolites in RE patients via integrated multi-omics (focusing on the gut microbiota-glutamine axis), and verify the activation status of this pathway in RE inflammatory models and the anti-inflammatory effect of its targeted inhibition.

**Methods:**

RE patients and healthy controls (HCs) were enrolled. Fecal metagenomic sequencing and serum untargeted metabolomics (LC-MS/MS) were performed to identify differential gut microbiota and serum metabolites between the two groups, followed by Pearson correlation analysis to explore their associations. *In vitro* experiments were conducted on human esophageal epithelial cells (HEECs) divided into four groups: normal, inflammatory, glutamine-supplemented, and inflammatory + glutamine + glutaminase inhibitor (BPTES) groups. qPCR was used to detect the mRNA expression of glutamine-glutamate pathway molecules (GLS, c-Myc, SLC1A5), mucosal barrier markers (ZO-1, Occludin), and pro-inflammatory cytokines (IL-8, IL-6, IL-1β, TNF-α). Intracellular concentrations of glutamine, glutamate, and α-ketoglutarate were measured, and the anti-inflammatory effect of BPTES was verified.

**Results:**

RE patients showed significant differences in gut microbiota diversity and composition compared with HCs, with *Bacteroidota*, *Pseudomonadota*, *Escherichia coli*, and *Klebsiella pneumoniae* as dominant taxa. Serum metabolomics revealed elevated glutamine and glutamate in RE patients, which were identified as key differential metabolites related to RE pathogenesis. Pearson analysis revealed that alterations in serum metabolite profiles of RE patients were significantly correlated with changes in gut bacterial abundance. Notably, glutamate-glutamate (Glu-Glu) metabolism exhibited negative correlations with multiple bacterial genera (*Acrocarpospora*, *Limnobacter*, *Pseudobacter*, *Shewanella*, and *Tropicimonas*). *In vitro*, inflammatory HEECs exhibited increased intracellular glutamine, glutamate, and α-ketoglutarate, upregulated glutamine-glutamate pathway molecules and pro-inflammatory cytokines, and downregulated mucosal barrier markers. Exogenous glutamine alone failed to alleviate inflammation, while combined with BPTES significantly reversed pathway activation and mitigated inflammation in inflammatory HEECs.

**Conclusion:**

RE patients exhibit significant gut microbiota dysbiosis (dominated by *Bacteroidota*, *Pseudomonadota*, *Escherichia coli*, and *Klebsiella pneumoniae*) and abnormal glutamine metabolism (elevated serum glutamine and glutamate). Pearson analysis reveals that the glutamine-glutamate pathway correlates negatively with multiple bacterial genera (*Acrocarpospora*, *Limnobacter*, *Pseudobacter*, *Shewanella*, and *Tropicimonas*). The glutamine-glutamate pathway is activated in inflammatory esophageal epithelial cells, and targeted GLS inhibition by BPTES reverses pathway activation and mitigates inflammation. These findings highlight the gut microbiota-glutamine axis as potential diagnostic biomarkers and therapeutic targets for RE, providing new insights into pathogenesis and a basis for novel clinical interventions.

## 1 Introduction

Gastroesophageal reflux disease (GERD) is a globally prevalent disorder with a prevalence rate of approximately 6%∼20% (Dunbar, 2024; Gupta, 2025). Reflux esophagitis (RE), an important subtype of GERD, is often severe and significantly impacts patients’ quality of life (Salimian et al., 2022). Characterized by esophageal mucosal hyperemia, erosion, and even ulceration due to persistent gastroesophageal reflux, RE manifests with refractory symptoms such as heartburn, regurgitation, and retrosternal pain (Domingues and Moraes-Filho, 2021; Maret-Ouda et al., 2021). Proton pump inhibitors (PPIs) remain the first-line therapy for RE; however, up to 30% of patients exhibit inadequate response or symptom recurrence, and long-term PPI use is associated with adverse effects including gut microbiota dysbiosis, nutritional deficiencies, and increased risk of enteric infections (Macke et al., 2020; Pasta et al., 2024). Notably, recent evidence indicates that long-term PPI administration triggers a “gut microbiota extinction storm,” characterized by a 47% reduction in α-diversity and a 9.4-fold increase in pathogenic bacterial colonization, which may further exacerbate esophageal mucosal inflammation through the gut-esophageal axis (Duysburgh et al., 2023; Fossmark and Olaisen, 2024). These limitations highlight the urgent need to elucidate the underpinning pathogenic mechanisms of RE beyond acid reflux and develop novel targeted therapeutic strategies.

Accumulating evidence has underscored the critical role of gut microbiota dysbiosis in the pathogenesis of GERD (Francis et al., 2023; Wang et al., 2024). The gut microbiota, a complex ecosystem interacting closely with the host, maintains gastrointestinal homeostasis by regulating mucosal barrier integrity, immune responses, and metabolic profiles (Zheng and Tao, 2025). Metagenomic studies have confirmed that GERD patients exhibit distinct microbial dysbiosis, typically characterized by enrichment of pathogenic bacteria such as *Escherichia coli* and *Klebsiella pneumoniae*, and depletion of beneficial butyrate-producing bacteria (Mozheiko et al., 2023). However, the downstream metabolic cascades through which gut microbiota dysbiosis drives esophageal inflammation remain poorly defined. Emerging studies have highlighted the role of gut microbial metabolites as key mediators in the gut-esophageal axis, but the specific metabolic pathways involved and their crosstalk with host metabolism in RE have not been systematically explored.

Glutamine, the most abundant free amino acid in humans, serves as a critical regulator of inflammation and mucosal barrier function, and its metabolic dysfunction has been implicated in various inflammatory gastrointestinal diseases (Cortes et al., 2022; Leite et al., 2025). The glutamine-glutamate metabolic pathway, a central branch of glutamine catabolism, mediates the conversion of glutamine to glutamate and α-ketoglutarate, which are essential for cellular energy supply and inflammatory signal transduction (Jin et al., 2024). Xiao et al. demonstrated that this pathway directly modulates mucosal barrier integrity in gastrointestinal diseases, suggesting its potential role in inflammatory pathogenesis (Xiao et al., 2024). For RE, preliminary serum metabolic profiling has revealed abnormal amino acid metabolism, including altered glutamine levels, but three key knowledge gaps remain: (1) whether the glutamine-glutamate pathway is abnormally activated in RE; (2) the association between this pathway and gut microbiota dysbiosis; and (3) whether targeted inhibition of this pathway exerts anti-inflammatory effects in RE. Moreover, the gut microbiota-glutamine axis has emerged as a novel regulatory hub in metabolic inflammation, but its relevance to RE remains unaddressed, representing a critical research gap.

To address these critical gaps, the present study employed an integrated multi-omics approach (fecal metagenomics and serum untargeted metabolomics) to systematically characterize the gut microbiota and metabolic profiles of RE patients, with a specific focus on the glutamine-glutamate metabolic pathway. We further validated the activation status of this pathway in RE inflammatory cell models and evaluated the anti-inflammatory efficacy of targeted glutaminase (GLS) inhibition. By exploring the crosstalk between gut microbiota and the glutamine-glutamate pathway, this study aims to clarify the role of the gut microbiota-glutamine axis in RE pathogenesis. The findings are expected to identify potential diagnostic biomarkers and novel therapeutic targets for RE, providing a theoretical basis for optimizing clinical management of this refractory disease.

## 2 Materials and methods

### 2.1 Subject selection

This study included 35 patients diagnosed with RE and 15 healthy controls (HCs) who were admitted to Tianjin Nankai Hospital, affiliated with Tianjin Medical University. Participants were aged between 35 and 84 years. The diagnosis of RE was based on endoscopic findings, evaluated using the Los Angeles (LA) classification, with specific criteria outlined in the “Lyon Consensus 2.0” (Gyawali et al., 2024). Healthy control participants were recruited from Tianjin Nankai Hospital. Exclusion criteria for all participants were: (1) use of proton pump inhibitors or gastrointestinal motility drugs within the week prior to enrollment; (2) structural abnormalities of the digestive tract; (3) eosinophilic esophagitis; (4) Helicobacter pylori infection; (5) metabolic disorders; (6) coagulation dysfunction; (7) functional diseases of the heart, liver, kidney, or other major organs; and (8) hematologic or immune system disorders; and (9) use of antibiotics, probiotics, or prebiotics within the two months preceding stool sample collection. General demographic information (age, sex, body mass index [BMI]), gastrointestinal symptoms, routine blood test results, gastroscopy findings, and 24-h esophageal pH monitoring results (for RE patients) were collected. Participants had not used antibiotics, probiotics, or prebiotics in the two months preceding stool sample collection (Ye et al., 2023).

### 2.2 Assessment and control of confounders

Given that diet, antibiotic history, and PPI exposure are well-documented strong determinants of gut microbiota composition, systematic assessment and rigorous control of these confounders were performed to ensure the reliability and robustness of the study results. All confounding factors were evaluated through standardized questionnaires, face-to-face interviews, and medical record reviews, with detailed screening and control strategies as follows.

For dietary assessment, all participants completed a validated semi-quantitative food frequency questionnaire (FFQ) to collect detailed information on dietary intake over the past 3 months. The FFQ included 12 major food categories, covering carbohydrates, proteins, fats, fruits, vegetables, fermented foods, dairy products, processed meats, staple foods, condiments, beverages, and snacks. The frequency and quantity of each food item consumed were recorded, and principal component analysis (PCA) was used to cluster dietary patterns. The differences in dietary composition between the RE group and HCs group were compared using statistical methods to exclude potential confounding effects of diet on gut microbiota composition.

Regarding antibiotic history, participants were first screened according to the exclusion criterion of no antibiotic use within 2 months before enrollment. To further avoid residual effects of antibiotics on gut microbiota, detailed antibiotic use history (including antibiotic type, duration of use, and dosage) over the past 6 months was collected through face-to-face interviews. Participants with a history of antibiotic use within 2–6 months before enrollment were excluded from the study. For the remaining eligible participants, the frequency, duration, and type of past antibiotic use were compared between the RE group and HC group to ensure no significant intergroup differences that could confound the study results.

For PPI exposure history, considering the significant impact of PPI use on gut microbiota structure, detailed PPI use information (including PPI type, dosage, and duration of use) was systematically collected through medical record review and self-reported questionnaires. Participants with PPI use within 1 month before enrollment were excluded. Participants with a history of PPI use more than 1 month prior to enrollment were stratified, and the proportion of such participants was compared between the two groups. Additionally, subgroup analysis was performed to verify whether past PPI use affected the main findings of gut microbiota dysbiosis in RE patients.

All data related to confounders were recorded in a standardized database, and statistical analyses were performed to confirm that there were no significant intergroup differences in dietary patterns, antibiotic history, and PPI exposure history (*P* > 0.05), ensuring that these confounders did not interfere with the validity of the study conclusions.

### 2.3 Fecal and blood sample collection

Stool samples and serum samples were collected for metagenomic sequencing analysis and untargeted metabolomics detection, respectively. According to the sample collection guidelines, fresh fecal samples and fasting venous blood samples were collected at Tianjin Nankai Hospital: fecal samples were collected using sterile collection tubes pre-filled with DNA stabilization solution to preserve microbial nucleic acids, immediately mixed thoroughly, transported on ice, and stored at −80 °C until DNA extraction; blood samples were collected using sterile vacuum blood collection tubes without anticoagulant, allowed to stand at room temperature for coagulation, centrifuged to separate serum, and the obtained serum was stored at −80 °C until subsequent detection. Clinical characteristics and demographic data of all participants were recorded at the time of sample collection. This study was conducted in accordance with the ethical principles outlined in the Declaration of Helsinki. The protocol was approved by the Ethics Committee of Tianjin Nankai Hospital (NKYY_YXKT__IRB_2023_063_01), and all participants provided written informed consent prior to enrollment.

### 2.4 Metagenomic sequencing

Sample processing and library construction: Fecal microbial DNA was extracted using HiPure Stool DNA Kits (Magen, Guangdong, China) according to manufacturer protocols. For sequencing library preparation, genomic DNA was sheared into ∼350 bp fragments via Covaris acoustic disruption, followed by end-polishing, dA-addition, and adapter ligation. Ligated products underwent PCR enrichment, size selection, and SPRI purification. Library quality was assessed through fluorometric quantification (Qubit 2.0), fragment analysis (Agilent 2100, target insert size 350 bp), and quantitative PCR (effective concentration > 3 nM). Qualified libraries were multiplexed and sequenced on Illumina NovaSeq platform generating 150 bp paired-end reads.

Bioinformatics analysis: Raw reads were quality-filtered using fastp (v0.20.0) with the following criteria: adapter contamination removal; exclusion of reads with > 50% low-quality bases (Q ≤ 5) or > 10% ambiguous nucleotides. Host-derived sequences were eliminated by mapping against the host with Bowtie2 (v2.3.5, parameters: –end-to-end –sensitive -I 200 -X 400). Clean reads were assembled *de novo* using MEGAHIT (v1.1.3, –presets meta-large). Resulting scaffolds were split at ambiguous bases (N) to generate scaftigs. ORF prediction was performed on scaftigs ≥ 500 bp using MetaGeneMark (default settings), discarding predictions < 100 nt. Redundancy removal employed CD-HIT (v4.8.1, -c 0.95 -aS 0.9 -g 1 -d 0) to establish a non-redundant gene catalog. Bowtie2 mapped sample reads to this catalog (–end-to-end –sensitive), filtering genes with ≤ 2 supporting reads. Gene abundance was computed as reads per kilobase (RPK). Taxonomic classification utilized DIAMOND (v0.9.24, blastx, e-value ≤ 1e-5) alignment against the Micro_NR database (NCBI NR subset: Bacteria, Archaea, Fungi, Viruses). The LCA algorithm (MEGAN) resolved ambiguous assignments. Species-level abundances were derived by summing gene abundances annotated to each taxon. β-diversity analyses incorporated PCA, PCoA, and NMDS; inter-group comparisons employed ANOSIM, MetaGenomeSeq, and LEfSe (LDA score > 4). Functional profiling aligned unigenes against KEGG, eggNOG, CAZy, VFDB, and PHI databases (DIAMOND, e-value ≤ 1e-5, best-hit selection). Antibiotic resistance determinants were identified through CARD database screening using RGI (v5.0, e-value < 1e-30). Mobile genetic elements were detected via ISFinder, Integrall, and plasmid database homology searches.

### 2.5 Metabolomics analysis

Sample preparation: Serum metabolites were recovered through protein precipitation with chilled organic solvent. Aliquots of 100 μL were vortex-mixed with 400 μL of ice-cold 80% methanol for 1 min, incubated on wet ice for 5 min, and clarified by centrifugation at 15,000 × *g* for 20 min at 4 °C. The resulting supernatants were diluted with LC-MS grade water to achieve 53% methanol content, re-centrifuged under identical conditions, and the final extracts were transferred to autosampler vials for instrumental analysis. To monitor analytical drift and exclude batch effects, pooled quality control specimens were generated by combining equivalent volumes from each biological sample and analyzed at regular intervals throughout the run sequence. Procedural blanks consisting of 53% aqueous methanol underwent identical processing to enable background subtraction and contaminant screening. All specimens were maintained at −80 °C prior to analysis and thawed once immediately before processing to preclude freeze-thaw degradation.

Chromatographic separation and mass spectrometric detection: Metabolic profiling was conducted on a Vanquish UHPLC platform interfaced with a Q Exactive HF hybrid quadrupole-Orbitrap mass spectrometer (both Thermo Fisher Scientific, Bremen, Germany). Chromatographic resolution was achieved using a Hypersil Gold C18 analytical column (100 mm × 2.1 mm, 1.9 μm particle size) maintained at 40 °C, with mobile phase delivered at 0.2 mL/min and sample injection volume of 5 μL. The autosampler tray temperature was held at 4 °C to preserve sample integrity. Gradient elution was programmed as follows: initial 2% B held for 1.5 min, linear increase to 85% B over 1.5 min, further ramp to 100% B by 10 min, maintained for 0.1 min, then reverted to 2% B at 10.1 min and equilibrated until 12.0 min. Eluent A consisted of 0.1% formic acid in water for positive electrospray ionization or 5 mM ammonium acetate (pH adjusted to 9.0) for negative mode; eluent B was methanol in both ionization polarities.

Mass spectrometric acquisition employed a heated electrospray ionization source (HESI-II) operating in polarity-switching mode. Source parameters included: spray voltage ± 3.5 kV, sheath gas 35 arbitrary units, auxiliary gas 10 L/min, capillary heater 320 °C, S-lens radio frequency amplitude 60, and auxiliary gas heater 350 °C. Survey full-scan MS spectra were acquired from m/z 100 to 1,500 at resolving power 70,000 (full width at half maximum, FWHM) with automatic gain control target of 3 × 10^6^ charges and maximum injection time 100 ms. Data-dependent tandem mass spectra were collected at 17,500 FWHM resolution using normalized collision energies of 20, 40, and 60 eV with AGC target 1 × 10^5^, maximum IT 50 ms, and dynamic exclusion of 10 s.

Data processing and metabolite identification: Raw spectra were processed through Compound Discoverer 3.3 (Thermo Fisher Scientific, 2019) implementing: (i) chromatographic alignment; (ii) feature detection (5 ppm tolerance, 30% intensity deviation, S/N > 3); (iii) adduct ion grouping; (iv) Genesis algorithm peak integration; and (v) QC-based normalization. Metabolite identification employed a tiered framework: elemental composition prediction (< 5 ppm), MS/MS matching against mzCloud, mzVault v2.0, and MassList v2019 (similarity > 80), and multidimensional validation (retention time ± 0.2 min, fragment ions). Relative quantification included TIC normalization, CV > 30% filtering, and log_2_ transformation. Multivariate and univariate analyses were conducted in metaX v1.4.6, R v3.4.3, and Python v2.7.6.

Putative metabolite identities were established through a tiered evidence framework. Elemental compositions were predicted from accurate mass measurements (deviation < 5 ppm from theoretical) and isotopic pattern distributions. Tandem mass spectral matching against three reference repositories—mzCloud (online spectral database),^1^ mzVault (v2.0, manufacturer-curated local library), and MassList (v2019, high-resolution MS collection)—provided structural candidates with similarity scores exceeding 80 points. Final annotations integrated retention time congruence ( ± 0.2 min tolerance), exact mass fidelity, and fragment ion interpretability, with ambiguous assignments subjected to manual curator review.

Quantification workflow and statistical mining: Relative quantification followed a standardized protocol: raw peak areas underwent QC-based drift correction using the first pooled injection as reference, followed by total ion current normalization where individual feature responses were divided by the aggregate ion signal and multiplied by the corresponding QC1 scaling factor. Stringent quality filtering eliminated: (i) metabolic features exhibiting coefficient of variation > 30% across QC replicates; (ii) signals present in blank injections exceeding 10% of mean biological sample intensity; and (iii) compounds detected in fewer than 50% of specimens within any experimental group. Missing values were imputed at half the minimum observed detection limit prior to log_2_ transformation for downstream statistical modeling.

Functional characterization leveraged three annotation resources: Kyoto Encyclopedia of Genes and Genomes (KEGG, 2020 release),^2^ Human Metabolome Database (HMDB v4.0),^3^ and LIPID MAPS Structure Database (2020 release).^4^ Chemometric analysis was orchestrated through metaX (v1.4.6) implementing unsupervised principal component analysis and supervised partial least squares discriminant analysis with VIP score computation. PLS-DA model robustness was assessed by permutation testing (200 iterations). Model validity was established when: (i) all R^2^ values exceeded corresponding Q^2^ values, and (ii) the Q^2^ regression line intercept was negative (< 0), indicating absence of overfitting. Univariate group comparisons employed Welch’s *t*-test with Benjamini-Hochberg false discovery rate adjustment. Differential metabolites satisfied simultaneous criteria of VIP > 1, FDR-adjusted *P* < 0.05, and fold change ≥ 2 or ≤ 0.5. Hierarchical clustering utilized z-score standardized abundances with Ward’s linkage, while pathway over-representation was assessed by hypergeometric testing (significance threshold *P* < 0.05). All statistical computing was performed in R (R version R-3.4.3), Python (Python 2.7.6 version) and CentOS (CentOS release 6.6).

### 2.6 Pearson correlation analysis

To investigate the linear covariation between gut microbiota and host metabolism, Pearson product-moment correlation analysis was performed to quantify associations between differentially abundant microbial taxa and annotated metabolites. The analysis focused on discriminant bacterial genera identified from metagenomic sequencing and fully characterized differential metabolites from UHPLC-MS/MS profiling (RE versus control comparison). Statistical significance was set at *P* < 0.05 (two-tailed), with correlation coefficients (*r*) categorized as weak (|*r*| = 0.10–0.30), moderate (|*r*| = 0.30–0.50), or strong (|*r*| > 0.50) linear relationships.

Correlation results were visualized through Sankey diagrams, with flow widths representing the strength of associations between key microbial genera (default Top 10) and differential metabolites (default Top 20), where red indicates positive correlations and blue indicates negative correlations.

All computations were executed in R software (version 3.4.3) using the ggalluvial package for Sankey diagram generation.

### 2.7 Cell culture

Human normal esophageal epithelial cells (HEEC) were maintained in complete Dulbecco’s Modified Eagle Medium (DMEM) fortified with 20% fetal bovine serum (FBS). Cell cultivation was performed in a controlled incubator environment set at 37 °C with 5% CO_2_ and saturated humidity, which provided a favorable microenvironment for supporting optimal cellular growth and proliferation.

### 2.8 CCK-8 assay

Cell viability was measured using CCK-8. HEEC cells (1 × 10^4^/well) were plated in 96-well plates and cultured for 24 h. Cells were then exposed to acidic medium (pH 5.0) with DCA (50–300 μM) for 2–8 h. After treatment, CCK-8 solution was added and incubated at 37 °C for 2 h. Absorbance at 450 nm was read using a microplate reader. Each condition was performed in triplicate.

### 2.9 Induction of inflammatory phenotype

To establish an inflammatory phenotype in HEEC, an inflammatory stimulation microenvironment was constructed. The culture medium was adjusted to pH 5.0 through acidification to recapitulate the acidic milieu present in pathological states. Simultaneously, the cells were subjected to treatment with 100 μM deoxycholic acid (DCA) to amplify the inflammatory response. This combined intervention of acidic pH exposure and DCA treatment was devised to simulate the characteristic pathological alterations of esophageal epithelial tissue under disease conditions.

### 2.10 Real-time quantitative PCR

Total RNA was isolated from cells and colon tissues using TRIzol reagent (Invitrogen) according to the manufacturer’s protocol. Complementary DNA (cDNA) was synthesized from RNA using the Hifair^®^ III First Strand cDNA Synthesis Kit (Yeasen Biotech). Quantitative real-time PCR (qRT-PCR) was conducted with the Hieff^®^ qPCR SYBR Green system and the LightCycler 480 SYBR Green I Master Mix (Roche) following the manufacturer’s guidelines. Gene expression levels were normalized to GAPDH and calculated using the 2^–ΔΔCt^ method. Primer sequences are provided in Supplementary Table 1.

### 2.11 Western blot analysis

Total protein was extracted from HEEC cells using RIPA lysis buffer supplemented with protease inhibitor cocktail (Roche, Switzerland) and phosphatase inhibitors. Protein concentration was determined by BCA assay (Thermo Fisher, USA). Equal amounts of protein (30 μg) were separated by 10% SDS-PAGE and transferred onto PVDF membranes (Millipore, USA). Membranes were blocked with 5% skim milk in TBST for 1 h at room temperature, then incubated overnight at 4 °C with primary antibodies: anti-GLS (1:10000, Proteintech, USA), anti-SLC1A5 (1:5000, Proteintech, USA), anti-ZO-1 (1:1000, CST, USA), anti-Occludin (1:1000, CST, USA), and anti-GAPDH (1:10000, Abways, USA). After washing with TBST, membranes were incubated with HRP-conjugated secondary antibodies (1:5000, Jackson ImmunoResearch, USA) for 1 h at room temperature. Protein bands were visualized using ECL chemiluminescence reagent (Thermo Fisher, USA) and captured by ChemiDoc imaging system (Bio-Rad, USA). Densitometric analysis was performed using ImageJ software.

### 2.12 Determination of metabolite contents

The contents of intracellular glutamine, glutamate, and α-ketoglutarate were determined using a biochemical kit method. Cells from each group were collected, washed with pre-cooled PBS, and then lysed on ice by adding a specific lysis buffer. The lysate was centrifuged at 12,000 × *g* and 4 °C for 10 min, and the supernatant was collected as the sample to be tested. Subsequently, the supernatant, standard solution, and working solution were added to a 96-well plate at the ratio specified in the instruction manual, mixed thoroughly, and incubated at 37 °C in the dark for a certain period. Finally, a microplate reader was used to measure the absorbance value of each well at a specific wavelength, and the concentrations of intracellular glutamine, glutamate, and α-ketoglutarate were calculated according to the standard curve.

### 2.13 Statistical analysis

All experimental data were subjected to statistical analysis using GraphPad Prism 9.0 software. Depending on the experimental design, either one-way or two-way analysis of variance (ANOVA) was employed for data comparison. All results were expressed as the mean ± standard error of the mean (SEM), and a *P*-value of less than 0.05 was deemed to indicate a statistically significant difference.

## 3 Results

### 3.1 Analysis of gut microbial diversity between RE patients and HCs

**FIGURE 1:**
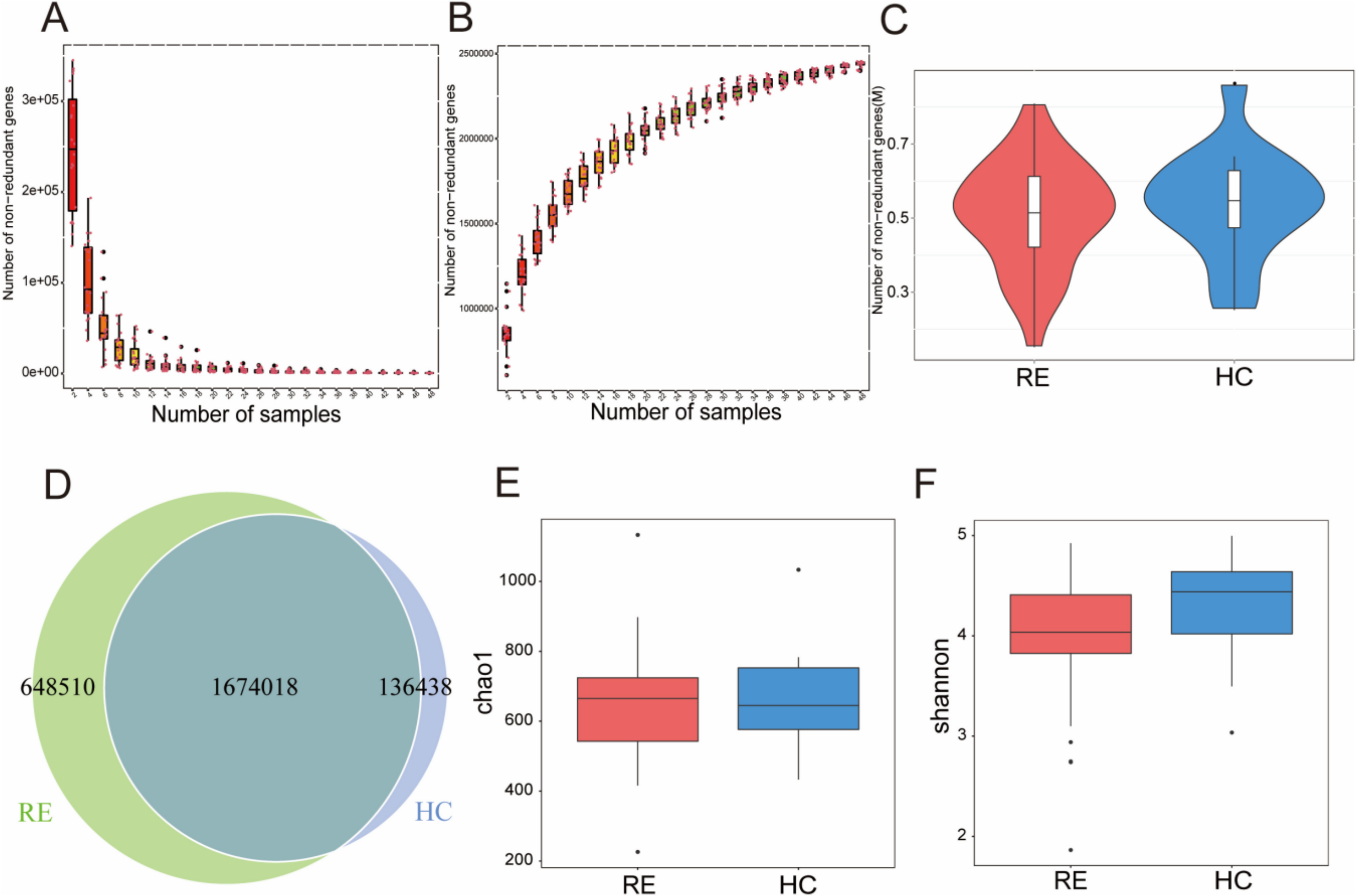
Analysis of gut microbial diversity between RE patients and HCs based on metagenomic sequencing. Metagenomic sequencing revealed significant ecosystem perturbations in RE patients (*n* = 35) compared to HCs (*n* = 15). **(A,B)** Core-pan gene analysis was performed to assess gene repertoire diversity across samples. Gene accumulation patterns were evaluated to ensure cohort-level sampling adequacy, with individual sample quality verified through independent thresholds (> 2 reads per gene) and host contamination filtering. **(C)** Marked reduction in total gene count indicates contracted functional repertoire of the gut microbiome in RE. **(D)** Venn diagram illustrates shared and unique gene pools, with RE-specific genes (648,510) substantially outnumbering HC-specific genes (136,438), suggesting dysbiotic community reconfiguration. **(E,F)** Significantly decreased α-diversity indices demonstrate loss of both richness and evenness in RE patients.

To investigate the characteristics of gut microbiota in patients with RE, we collected fecal samples from 35 individuals diagnosed with RE and 15 HCs. The median age in the RE group was 62 years (range: 35–84), with 45.71% male participants, while the HC group had a median age of 60 years (range: 30–80), with 33.33% male participants. BMI was marginally elevated in RE patients [median 24.6 kg/m^2^ (range: 21.3–28.5)] compared to HCs [23.2 kg/m^2^ (20.5–26.8)] (*P* = 0.312). Lifestyle factors including smoking, alcohol consumption, high-fat diet, spicy food intake, and caffeine consumption showed no significant differences between groups (*P* > 0.05), though trends toward higher prevalence of dietary risk factors were observed in the RE group. The most commonly reported symptoms in RE patients included regurgitation (94.29%), heartburn or chest pain (85.71%), vomiting (74.29%), and respiratory symptoms (40.00%). Notably, PPI use was significantly more prevalent in RE patients [28 (80.0%)] compared to HCs [0 (0.0%)] (*P* < 0.001). Antibiotic and probiotic use in the past 3 months was low and comparable between groups (*P* > 0.05) (Table 1). To assess differences in gut microbiota composition between RE patients and HCs, metagenomic sequencing was performed on fecal samples from both groups. Rarefaction analysis of the pan-genome curve (cumulative gene intersection of 50 samples) and the core-gene curve (gene intersection across all samples) demonstrated saturation plateauing, confirming adequate sequencing depth for comparative analysis (Figures 1A,B). Individual sample quality was independently verified through read depth thresholds (> 2 reads per gene) and host contamination filtering prior to downstream analyses. A box plot comparing gene counts revealed that the total number of genes in the microbiota of RE patients was lower than that in HCs (Figure 1C). Venn diagram analysis showed that 1,674,018 genes were shared between the two groups, with 648,510 genes unique to the RE group and 136,438 genes unique to the HC group (Figure 1D).

**TABLE 1:** Comparison of clinical features between RE group and healthy control group.

Variable	Healthy control group (*n* = 15)	RE group (*n* = 35)	*P*
Demographics			
Age media (range)	59 (31∼79)	63 (36∼83)	0.582
Male, *n* (%)	6 (40.00%)	15 (42.86%)	0.473
Female, *n* (%)	9 (60.00%)	20 (57.14%)	
BMI, median (range), kg/m^2^	23.2 (20.5–26.8)	24.6 (21.3–28.5)	0.312
Lifestyle factors			
Smoking, *n* (%)	2 (13.3)	6 (17.1)	0.728
Alcohol consumption, *n* (%)	2 (13.3)	5 (14.3)	0.917
High-fat diet, *n* (%)	3 (20.0)	12 (34.3)	0.286
Spicy food intake, *n* (%)	2 (13.3)	10 (28.6)	0.214
Caffeine intake (> 2 cups/day), *n* (%)	3 (20.0)	9 (25.7)	0.632
Main symptom reported, n (%)			
Chronic gastritis	0 (0%)	21 (60.00%)	
Heartburn or chest pain	0 (0%)	30 (85.71%)	
Regurgitation	0 (0%)	33 (94.29%)	
Vomiting	0 (0%)	26 (74.29%)	
Respiratory symptoms	0 (0%)	14 (40.00%)	
Medication use			
PPI use, *n* (%)	0 (0.0)	28 (80.0)	< 0.0001
Antibiotic use (past 3 months), *n* (%)	1 (6.7)	4 (11.4)	0.583
Probiotic use, *n* (%)	1 (6.7)	3 (8.6)	0.785

Data presented as median (range) for continuous variables and *n* (%) for categorical variables. *P*-values calculated by Mann–Whitney U test (continuous) or chi-square/Fisher’s exact test (categorical). BMI, body mass index; PPI, proton pump inhibitor.

Microbial diversity within the gut community was evaluated using Chao1, Observed species, Shannon, and Simpson indices. The Chao1 and Observed species indices reflect species richness, whereas the Shannon and Simpson indices reflect species diversity. Our results revealed a significant alteration in α-diversity in RE patients compared to HCs (Figures 1E,F and Supplementary Figures 1A,B). Collectively, these findings indicate the presence of gut microbial dysbiosis in RE patients.

### 3.2 Analysis of gut microbial abundance between RE patients and HCs

The community structure composition diagram illustrates the microbial compositions of different samples or groups across various taxonomic levels.

**FIGURE 2:**
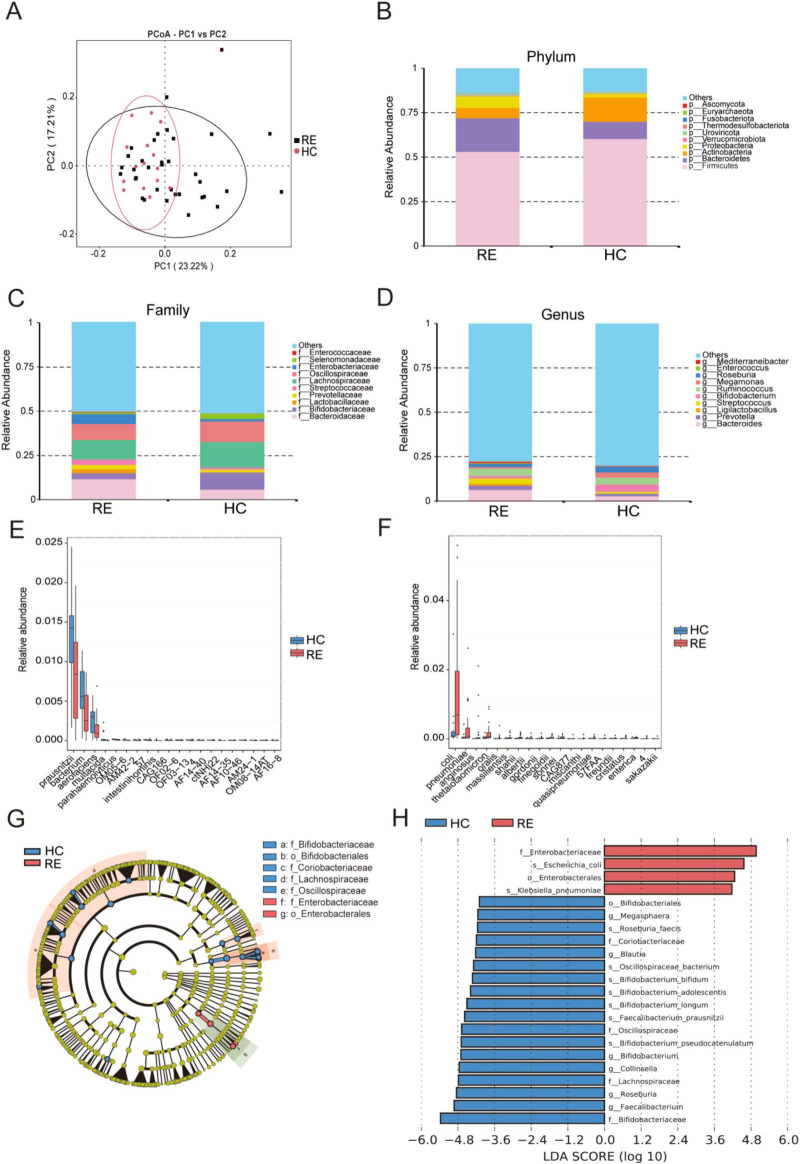
Gut microbiota differences based on metagenomic sequencing. Comparative metagenomic profiling demonstrates distinct community assembly patterns between RE patients and HCs across taxonomic hierarchies. **(A)** PCoA based on Bray-Curtis distance reveals distinct clustering of RE and HC samples, confirming community-level divergence. **(B–D)** Relative abundance analysis shows elevated *Bacteroidota* and *Pseudomonadota* at phylum level, expansion of *Bacteroidaceae*, *Prevotellaceae*, *Streptococcaceae*, and *Enterobacteriaceae* at family level, and increased *Bacteroides*, *Ligilactobacillus*, *Prevotella*, *Streptococcus*, and *Ruminococcus* at genus level in RE patients. Concomitantly, beneficial taxa including *Bifidobacteriaceae*, *Lachnospiraceae*, *Oscillospiraceae*, and genera *Bifidobacterium*, *Megamonas*, *Roseburia* exhibit marked depletion. Taxonomic annotations in this figure are based on the Genome Taxonomy Database (GTDB). Panel **(A)** (phylum level) includes taxa from Bacteria, Archaea, Fungi, Viruses, and other unclassified or minor groups. Panels **(B)** (family level) and **(C)** (genus level) include only bacterial taxa. The taxonomic groups and their corresponding colors, abbreviations, and display specifications are as follows: Bacteria: Including p_Firmicutes, p_Bacteroidetes, p_Actinobacteria, p_Proteobacteria, p_Fusobacteriota, p_Thermodesulfobacteriota, and p_Verrucomicrobiota; Archaea: p_Euryarchaeota; Fungi: p_Ascomycota; Viruses: p_Uroviricota; Others: unclassified or minor taxonomic groups (Q < 0.05). **(E,F)** Species-level differential analysis identifies significant upregulation of *Escherichia coli*, *Klebsiella pneumoniae*, and *Bacteroides* thetaiotaomicron, alongside downregulation of Faecalibacterium prausnitzii and *Bifidobacterium* adolescentis in RE (Q < 0.05). **(G,H)** LEfSe analysis (LDA > 4) confirms *E. coli* and *K. pneumoniae* as RE-enriched biomarkers, with *Bifidobacterium* and Faecalibacterium as HC-enriched protective indicators.

PCoA demonstrated a distinct separation in gut microbiota composition between the two groups (Figure 2A).

At the phylum level, the gut microbiota of the HC group was predominantly composed of *Bacillota*, *Bacteroidota*, *Actinomycetota*, and *Pseudomonadota*. Compared with the HC group, the RE group exhibited a higher relative abundance of *Bacteroidota* and *Pseudomonadota*, while the relative abundance of *Bacillota* and *Actinomycetota* was decreased (Figure 2B).

At the family level, the gut microbiota in the HC group was mainly comprised of *Bacteroidaceae*, *Bifidobacteriaceae*, *Lactobacillaceae*, *Prevotellaceae*, *Streptococcaceae*, *Lachnospiraceae*, *Oscillospiraceae*, *Enterobacteriaceae*, and *Selenomonadaceae*. In the RE group, the relative abundances of *Bacteroidaceae*, *Prevotellaceae*, *Streptococcaceae*, and *Enterobacteriaceae* were significantly increased, whereas the abundances of *Bifidobacteriaceae*, *Lachnospiraceae*, *Oscillospiraceae*, and *Selenomonadaceae* were significantly decreased (Figure 2C).

At the genus level, the dominant genera in the HC group included *Bacteroides*, *Ligilactobacillus*, *Prevotella*, *Streptococcus*, *Ruminococcus*, *Bifidobacterium*, *Megamonas*, and *Roseburia*. In the RE group, the relative abundances of *Bacteroides*, *Ligilactobacillus*, *Prevotella*, *Streptococcus*, and *Ruminococcus* were significantly elevated, while those of *Bifidobacterium*, *Megamonas*, and *Roseburia* were markedly reduced (Figure 2D).

At the species level, the relative abundances of Faecalibacterium prausnitzii, *Bacteroides* bacterium, and *Bifidobacterium* adolescentis were significantly downregulated in the RE group compared with the HC group (Figure 2E), while the levels of *Escherichia coli*, *Klebsiella pneumoniae*, and *Bacteroides* thetaiotaomicron were significantly upregulated (Figure 2F).

LEfSe analysis was performed to identify significantly different microbial biomarkers between the two groups. The results indicated that *Escherichia coli* and *Klebsiella pneumoniae* were the most enriched species in the RE group, whereas *Bifidobacterium* and Faecalibacterium were more abundant in the HC group (Figures 2G,H).

### 3.3 Functional changes in the gut microbiota in patients with RE

**FIGURE 3:**
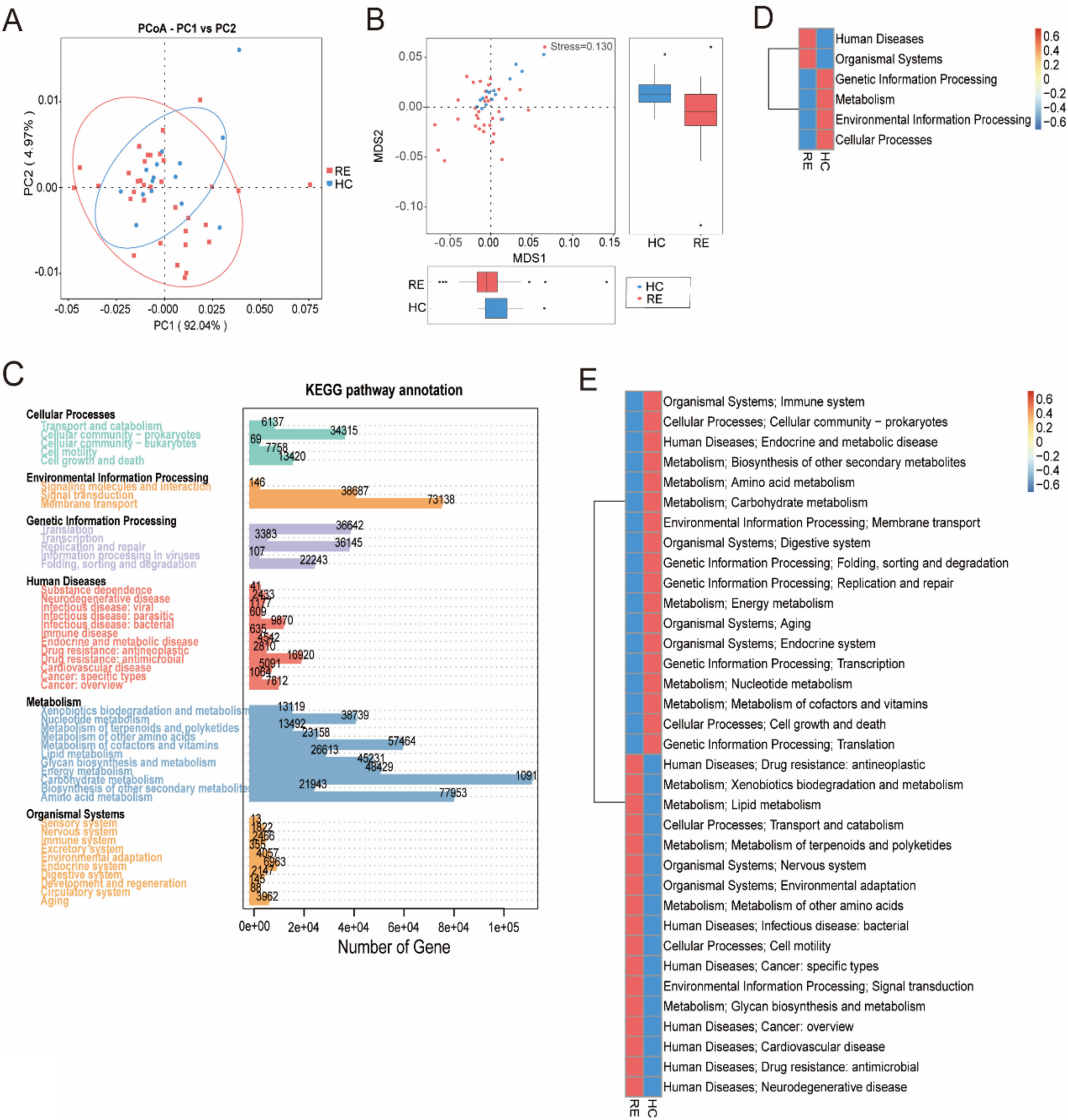
KEGG functional annotation of the gut microbiota genome. Based on KEGG functional annotation, significant divergence in microbial metabolic potential is revealed between RE patients and HCs. **(A,B)** Multivariate ordination (PCoA and NMDS) based on Bray-Curtis distance demonstrates distinct functional clustering, indicating ecosystem-level metabolic rewiring. NMDS stress = 0.130 (< 0.2, acceptable representation). **(C)** Gene count distribution reflects altered functional capacity. **(D,E)** Z-score standardized heatmap of differentially abundant KEGG pathways (Levels 1–2) highlights enrichment of carbohydrate metabolism, amino acid metabolism, and membrane transport functions in RE, concomitant with reduced cofactor and vitamin metabolism (color scale: –0.6 to +0.6, red = RE enrichment, blue = HC enrichment).

To explore the functional characteristics of the gut microbiota in RE patients, we further annotated the metagenomic data using the KEGG database. PCoA and NMDS based on KEGG modules demonstrated significant differences in the distribution of microbial functions between the RE and HC groups (Figures 3A,B). Gene count statistics and functional relative abundance analysis revealed that the most enriched functional categories were carbohydrate metabolism, membrane transport, amino acid metabolism, and the metabolism of cofactors and vitamins (Figures 3C–E).

Functional annotation using the eggNOG orthologous group (OG) database also showed a clear separation between the RE and HC groups. Both PCoA and NMDS analyses based on eggNOG modules indicated significant differences in microbial functional profiles between the two groups (Supplementary Figures 2A,B). The most abundant functional categories were replication, recombination and repair; transcription; cell wall/membrane/envelope biogenesis; carbohydrate transport and metabolism; and amino acid transport and metabolism (Supplementary Figures 2C,D,G).

Compared with the HC group, the RE group exhibited a marked downregulation of ABC transporters, carbohydrate transport proteins, and ATPases associated with various cellular activities (Supplementary Figure 2E). Conversely, genes associated with bacterial membrane components, conserved bacterial proteins, and cellular redox homeostasis were significantly upregulated in the RE group (Supplementary Figure 2F).

The CAZy enzyme classification (EC) profiles in the RE group were also significantly different from those in the HC group. PCoA and NMDS analyses based on CAZy modules demonstrated notable differences in microbial functional distribution between the RE and HC groups (Supplementary Figures 3A,B). Functional relative abundance analysis revealed that the most enriched categories were glycoside hydrolases, glycosyl transferases, and carbohydrate-binding modules (Supplementary Figures 3C–E,H).

Compared with the HC group, the RE group showed a marked downregulation in the abundance of enzymes such as endo-β-1,3-1,4-glucanase (EC 3.2.1.73), β-glucosylceramidase (EC 3.2.1.45), and amylomaltase/4-α-glucanotransferase (EC 2.4.1.25), which are involved in various cellular processes (Supplementary Figure 3F). Conversely, the levels of 1,2-diacylglycerol 3-glucosyltransferase (EC 2.4.1.157) and other enzymes (e.g., EC 2.4.1.26) were significantly upregulated in the RE group (Supplementary Figure 3G).

KEGG functional analysis suggested that the gut microbiota in RE patients may influence carbohydrate and amino acid metabolism, thereby altering the production of key metabolites and potentially impacting host energy homeostasis and immune regulation. Moreover, the enhanced membrane transport capacity may reflect microbial adaptation to nutrient exchange and metabolism in the intestinal environment. Shifts in the metabolism of cofactors and vitamins may also play a role in modulating host–microbiota symbiosis.

Further eggNOG analysis revealed disruptions in DNA repair and signal transduction functions, which could be associated with impaired esophageal epithelial repair mechanisms. CAZy analysis indicated that changes in polysaccharide-degrading enzymes and glycoside hydrolases might impact microbial metabolite production, potentially disrupting the gut–esophagus axis. Collectively, these findings suggest that gut microbiota dysbiosis may play a crucial role in the pathogenesis of RE, offering new insights into its underlying mechanisms and potential therapeutic targets.

### 3.4 Abnormal metabolic patterns in patients with RE

**FIGURE 4:**
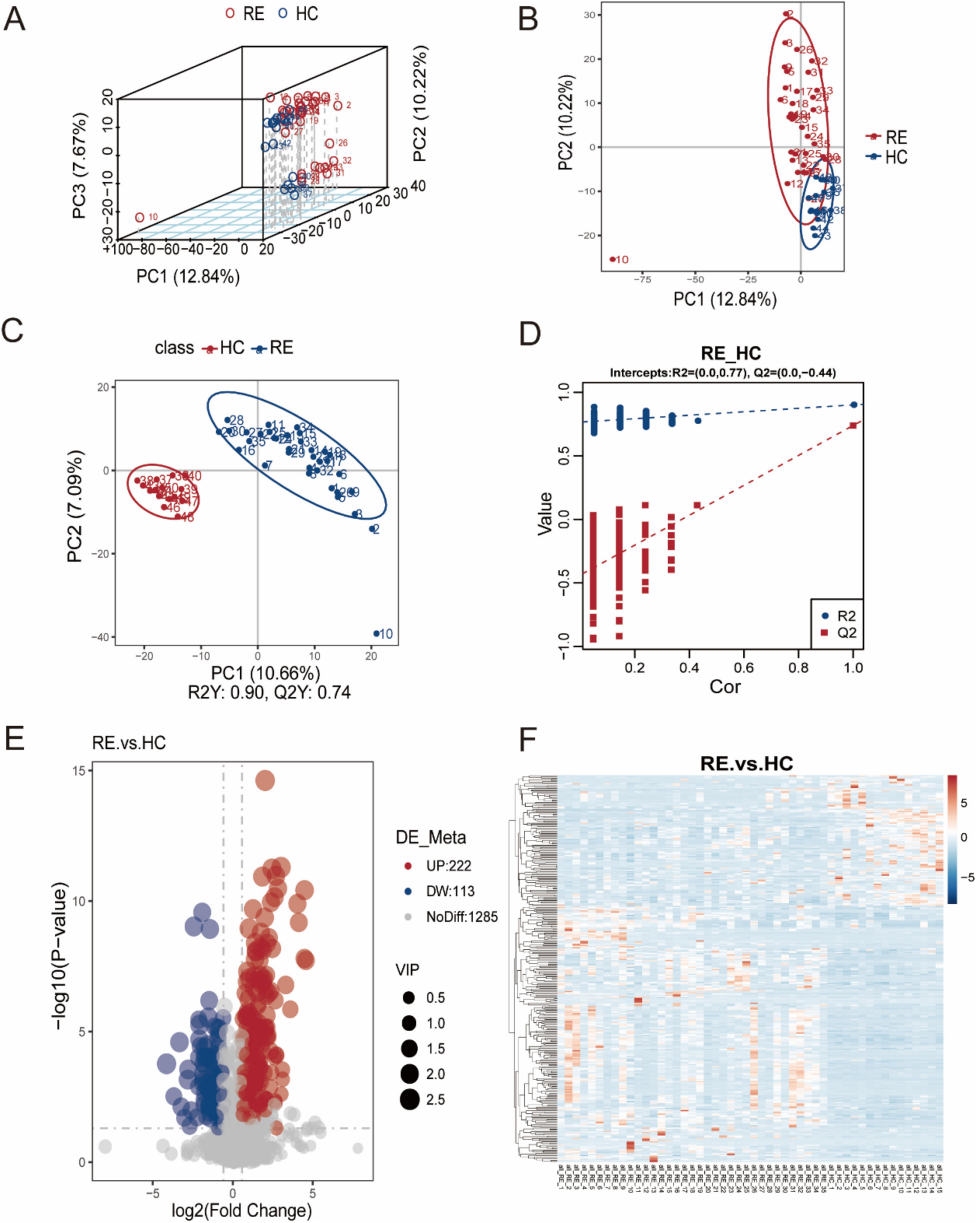
Serum metabolomics analysis of the groups. Untargeted LC-MS/MS profiling reveals distinct metabolic phenotypes between RE patients and HCs. **(A,B)** PCA demonstrates clear separation of serum metabolite profiles, indicating substantial metabolic reprogramming in RE. PC1, PC2, and PC3 explain 12.84, 10.22, and 7.67% of variance, respectively. **(C)** PLS-DA score plot showing RE-HC separation (R^2^Y = 0.90, Q^2^Y = 0.74). **(D)** Permutation test validation (*n* = 200): Q^2^ intercept = –0.44 (< 0, no overfitting); R^2^ intercept = 0.77. **(E)** Volcano plot illustrates 222 upregulated and 113 downregulated metabolic features in RE. **(F)** Hierarchical clustering reveals distinct metabolite signatures segregating RE from controls.

Building on the aforementioned metagenomic functional annotation findings, this study further conducted metabolomic profiling of serum samples from RE patients. Serum samples from both groups were analyzed using liquid chromatography-mass spectrometry (LC/MS), followed by pattern recognition through PCA and partial least squares-discriminant analysis (PLS-DA). The PCA results demonstrated a clear separation of metabolites between RE patients and control subjects, indicating substantial metabolic differences between the two groups (Figures 4A,B). Subsequently, PLS-DA was employed to screen for differential metabolites. The fitted PLS-DA model demonstrated strong explanatory power with R^2^Y = 0.90 and acceptable cross-validation with Q^2^Y = 0.74, suggesting apparent metabolic discrimination between RE and HC groups. However, model validation through permutation testing yielded a negative Q^2^ intercept of −0.44, significantly lower than the threshold of 0.05 for a valid predictive model, indicating overfitting and limited generalizability despite apparent class separation (Figures 4C,D). Specifically, 222 differential metabolic ions were upregulated and 113 were downregulated in the RE group compared with the HC group (Figure 4E). Hierarchical clustering analysis of these significant differential metabolites was performed and presented as heatmaps, which revealed distinct clustering patterns between the two groups (Figure 4F).

**FIGURE 5:**
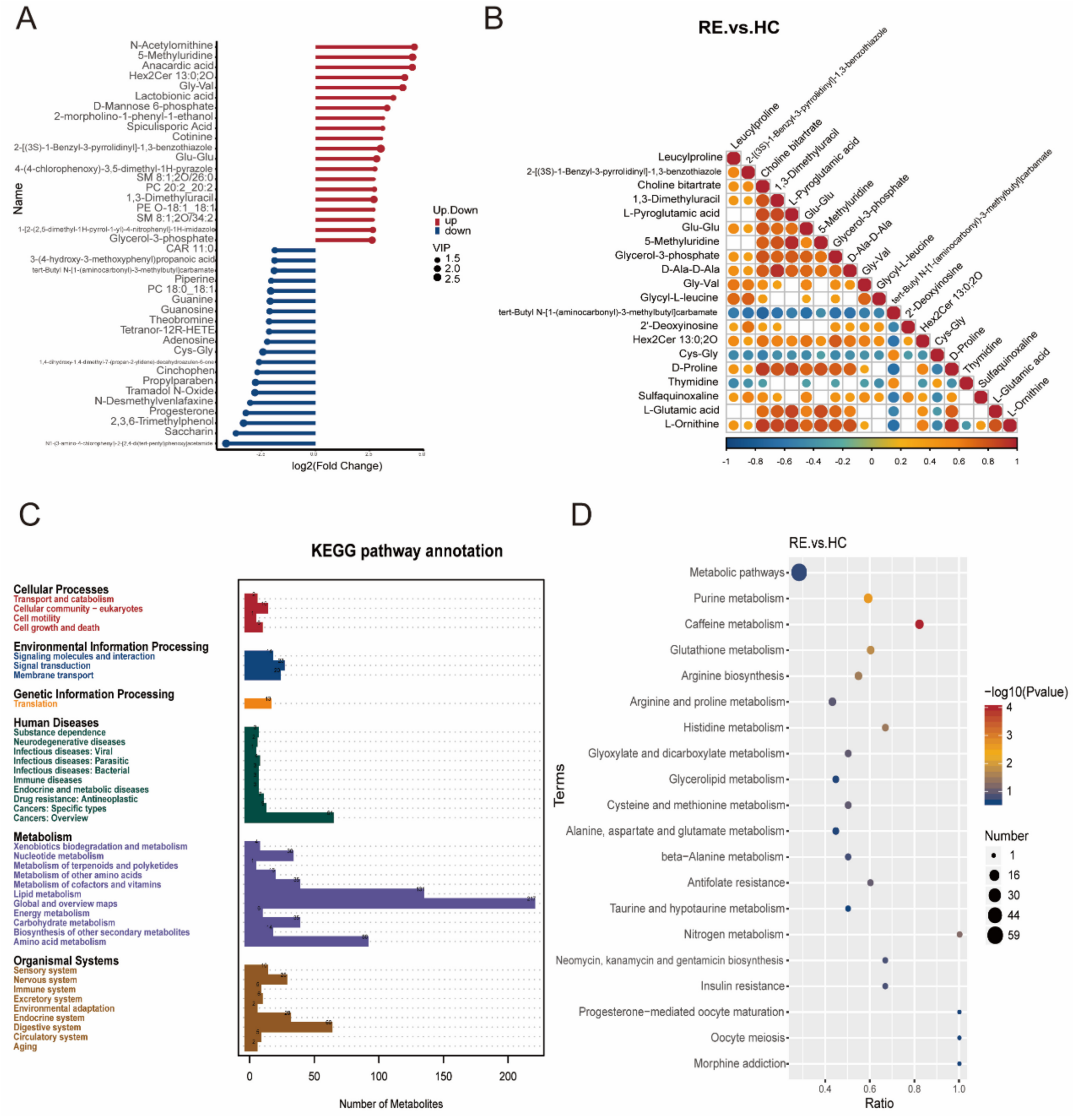
Aberrant metabolic patterns in RE patients. Differential metabolite profiling reveals disease-associated metabolic rewiring, with differential metabolites identified using univariate criteria (Welch’s *t*-test, FDR Q < 0.05) with fold change ≥ 2 or ≤ 0.5. **(A)** Top-ranked metabolites by fold change highlight amino acid derivatives and nucleotide metabolites as major discriminants. **(B)** Correlation network analysis demonstrates coordinated dysregulation among differential metabolites, suggesting pathway-level perturbations. **(C,D)** KEGG pathway mapping and bubble plot visualization identify global metabolism, amino acid metabolism (glutamate, arginine), and purine metabolism as significantly enriched pathways. Metabolite identification was based on mzCloud, mzVault, and MassList databases (similarity > 80, mass error < 5 ppm, retention time tolerance ± 0.2 min).

The top 20 metabolites, ranked by log_2_-transformed fold change, are displayed in a matchstick plot (Figure 5A). Levels of N-Acetylornithine, 5-Methyluridine, Anacardic acid, Glu-Glu, and Gly-Val were significantly upregulated in the RE group, whereas Piperine, Guanine, Guanosine, Cys-Gly, Tramadol N-Oxide, and Adenosine were downregulated. To explore the potential interactions among differentially abundant metabolites between the RE and HC groups, a correlation heatmap was constructed. The results revealed that Glu-Glu exhibited a positive correlation with both Choline bitartrate and 1,3-dimethyluracil. Specifically, the correlation coefficients indicated a significant positive association between Glu-Glu and Choline bitartrate, as well as between Glu-Glu and 1,3-Dimethyluracil (Figure 5B). This positive correlation pattern suggests that these metabolites may be involved in the same metabolic pathway or regulatory network, which might collectively contribute to the pathogenesis of RE. KEGG pathway enrichment analysis of the differential metabolites showed that the top three enriched categories were global and overview maps, amino acid metabolism, and lipid metabolism (Figure 5C). A bubble chart was used to visualize the top 20 pathways sorted by enrichment significance. Specifically, the metabolic discrepancies between patient with RE and HC group were mainly associated with the following pathways: purine metabolism, caffeine metabolism, glutathione metabolism, arginine biosynthesis, alanine, aspartate and glutamate metabolism (Figure 5D).

### 3.5 Correlation analysis between the gut microbe and metabolite levels

**FIGURE 6:**
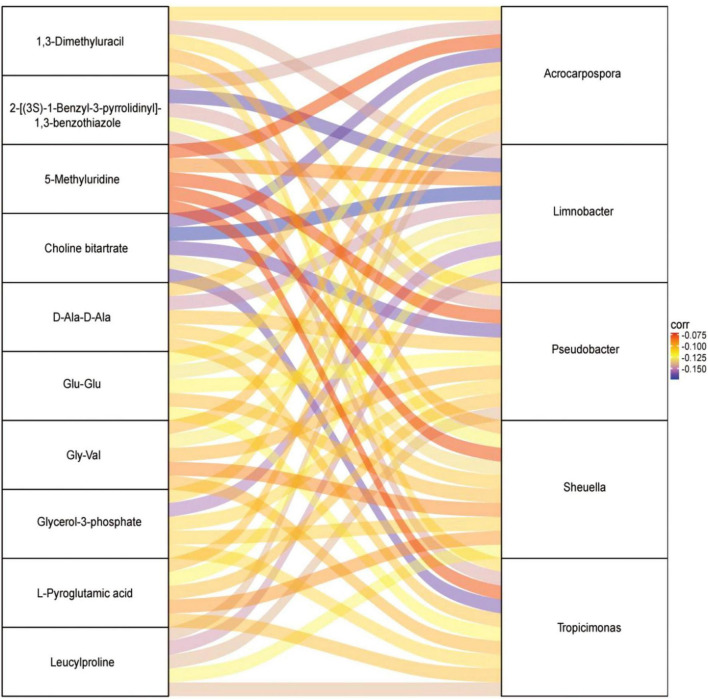
Correlation intensity between fecal microbiota and serum metabolites. Correlation intensity between fecal microbiota and serum metabolites. Pearson correlation analysis reveals significant associations between differentially abundant fecal bacterial genera (top 10 by *P*-value) and discriminant serum metabolites (top 20 by *P*-value) in RE (*P* < 0.05). Sankey diagram illustrates directional flow of correlations, with ribbon width proportional to correlation strength (red, positive; blue, negative). All statistical computations were executed in R software (version 3.4.3) using the ggalluvial package. Key associations highlight glutamate (Glu) coordination with multiple bacterial genera, supporting microbial metabolic input to host amino acid metabolism.

To clarify the association between gut microbiota and metabolites, Pearson correlation analysis was performed on the fully annotated common differential metabolites and key differential gut microbiota taxa identified from preliminary screening. Benjamini-Hochberg correction was applied to control for multiple testing. The results showed that alterations in serum metabolite profiles of RE patients were significantly correlated with changes in the abundance of various gut bacterial taxa. Specifically, Glu-Glu exhibited a negative correlation with multiple gut microbes (*Acrocarpospora*, *Limnobacter*, *Pseudobacter*, *Shewanella*, *Tropicimonas*) (Figure 6). These results indicate that there is a close interaction between the gut microbiota and metabolites, and this interaction may affect the pathogenesis of RE.

### 3.6 Inflammatory microenvironment promotes the activation of glutamine metabolic pathway in HEECs

To verify whether the glutamine metabolic pathway is activated in inflamed HEECs associated with RE, we first compared the differences in glutamine metabolism-related indexes between normal and inflamed HEECs. The inflamed HEEC model was established by acid (pH 5.0) combined with DCA stimulation, and the expression levels of glutamine metabolism pathway-related genes (GLS, c-Myc, SLC1A5) and the contents of key metabolites (glutamine, glutamate, α-ketoglutarate) in cells were detected.

**FIGURE 7:**
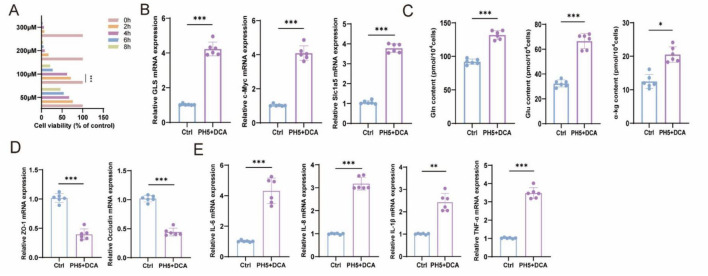
Activation of glutamine-glutamate pathway and its association with epithelial inflammation and barrier dysfunction in inflammatory HEECs. **(A)** HEECs viability was detected by CCK-8 assay after treatment with acidic DCA (50, 100, 200, 300 μM) for 2, 4, 6, and 8 h, with 0 h as the control. **(B)** mRNA expression of key molecules in glutamine-glutamate pathway; **(C)** Intracellular contents of glutamine, glutamate and α-ketoglutarate (pmol/10^4^ cells); **(D)** mRNA expression of tight junction-related genes; **(E)** mRNA expression of pro-inflammatory cytokines. *N* = 6 independent experiments, data are shown as the mean ± SEM; *P < 0.05, **P < 0.01, and ***P < 0.001; one-way ANOVA and a subsequent Tukey test was used.

We first established an inflammatory model using HEECs by screening various stimuli. Co-exposure to pH 5.0 and 100 μM deoxycholic acid (DCA) for 4 h proved optimal for eliciting a robust inflammatory response, as reflected by diminished cell viability (Figure 7A). qPCR results showed that compared with normal HEECs, the mRNA expression levels of GLS, c-Myc, and SLC1A5 in inflamed HEECs were significantly upregulated (Figure 7B). Meanwhile, metabolite detection results indicated that the intracellular contents of glutamine, glutamate and α-ketoglutarate in inflamed HEECs were all significantly increased (Figure 7C). These results suggested that the glutamine-glutamate metabolic pathway was activated in inflamed HEECs.

We further detected the expression of tight junction-related genes (ZO-1, Occludin) and inflammatory factors (IL-6, IL-8, IL-1β, TNF-α) to explore the correlation between glutamine pathway activation and epithelial inflammation. The results showed that compared with normal HEECs, the mRNA expression levels of ZO-1 and Occludin in inflamed HEECs were significantly downregulated (Figure 7D), while the mRNA expression levels of IL-6, IL-8, IL-1β, and TNF-α were significantly upregulated (Figure 7E), indicating that the activation of glutamine metabolic pathway was accompanied by enhanced inflammation and impaired epithelial barrier function in HEECs.

### 3.7 GLS inhibitor combined with glutamine inhibits inflammation in inflamed HEECs and reverses abnormal activation of glutamine metabolic pathway

To further investigate the regulatory role of glutamine in reflux esophagitis-associated epithelial inflammation, four experimental groups were established: normal HEECs group, inflamed HEECs group, inflamed HEECs + exogenous Gln group, and inflamed HEECs + exogenous Gln + GLS inhibitor group. The effects of exogenous Gln supplementation alone or in combination with GLS inhibitor on glutamine metabolic pathway activity, inflammatory response, and epithelial barrier function in inflamed HEECs were evaluated by detecting the expression of relevant genes and the levels of key metabolites.

**FIGURE 8:**
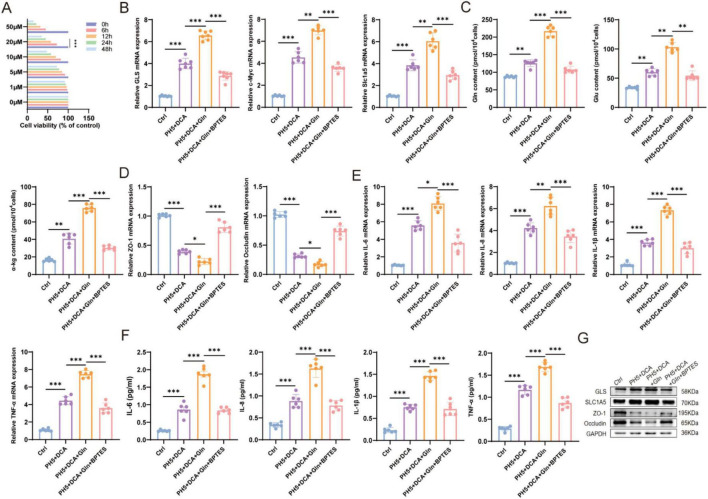
Effects of exogenous glutamine and glutaminase inhibitor on glutamine metabolism pathway, epithelial barrier function and inflammatory response in inflamed HEECs. **(A)** Cells were treated with various concentrations of BPTES (0, 1, 5, 10, 20, 50 μM) for indicated time periods (0, 6, 12, 24, 48 h), and cell viability was assessed by CCK-8 assay (*n* = 3). **(B)** mRNA expression of key molecules in glutamine-glutamate pathway; **(C)** Intracellular contents of glutamine, glutamate and α-ketoglutarate (pmol/10^4^ cells); **(D)** mRNA expression of tight junction-related genes; **(E)** mRNA expression of pro-inflammatory cytokines. **(F)** HEEC cells were stimulated with PH5 + DCA in the presence or absence of glutamine (Gln, 4 mM) and BPTES (20 μM) for 24 h. Cell culture supernatants were collected and levels of IL-6, IL-8, IL-1β, and TNF-α were measured by ELISA. **(G)** HEEC cells were stimulated with PH5 + DCA in the presence or absence of glutamine (Gln, 4 mM) and BPTES (20 μM) for 24 h. Total protein was extracted and expression levels of glutamine metabolism-related proteins (GLS, SLC1A5) and tight junction proteins (ZO-1, Occludin) were analyzed by Western blot. GAPDH was used as loading control. Glutamine supplementation upregulated GLS and SLC1A5 expression while downregulating tight junction proteins, effects that were reversed by BPTES treatment. *N* = 6 independent experiments, data are shown as the mean ± SEM; *P < 0.05, **P < 0.01, and ***P < 0.001; one-way ANOVA and a subsequent Tukey test was used.

Cells were treated with various concentrations of BPTES (0, 1, 5, 10, 20, 50 μM) for indicated time periods (0, 6, 12, 24, 48 h), and cell viability was assessed by CCK-8 assay (*n* = 3). Treatment with 20 μM BPTES for 24 h was selected as the optimal condition, yielding a moderate reduction in cell viability without inducing severe cytotoxicity (Figure 8A). qPCR results revealed that, compared with the inflamed HEECs group, the mRNA expression levels of GLS, c-Myc, and SLC1A5 were significantly upregulated in the inflamed HEECs + exogenous Gln group. In contrast, combined treatment with GLS inhibitor significantly downregulated the mRNA expression of these three genes compared to the inflamed HEECs + exogenous Gln group (Figure 8B). Metabolite detection showed that, relative to the inflamed HEECs group, the levels of glutamine, glutamate, and α-ketoglutarate were significantly increased in the inflamed HEECs + exogenous Gln group. In the inflamed HEECs + exogenous Gln + GLS inhibitor group, the levels of glutamate and α-ketoglutarate were significantly decreased compared with the inflamed HEECs + exogenous Gln group, while the glutamine level was further elevated (Figure 8C). These findings indicated that exogenous Gln supplementation could further activate the glutamine metabolic pathway in inflamed HEECs, and GLS inhibitor could effectively block this activation even in the presence of exogenous Gln.

For epithelial barrier function, compared with the inflamed HEECs group, the mRNA expression levels of ZO-1 and Occludin were significantly downregulated in the inflamed HEECs + exogenous Gln group. However, in the inflamed HEECs + exogenous Gln + GLS inhibitor group, the mRNA expression levels of ZO-1 and Occludin were significantly upregulated compared with the inflamed HEECs + exogenous Gln group (Figure 8D), showing no significant difference from those of the normal HEECs group. Regarding the inflammatory response, compared with the inflamed HEECs group, the mRNA expression levels of inflammatory factors (IL-6, IL-8, IL-1β, and TNF-α) were significantly upregulated in the inflamed HEECs + exogenous Gln group. After combined treatment with GLS inhibitor, the mRNA expression of these inflammatory factors was significantly downregulated compared to the inflamed HEECs + exogenous Gln group (Figure 8E), and was comparable to that of the normal HEECs group. These transcriptional alterations were further validated at the protein level by ELISA, which demonstrated concordant changes in the secretion of IL-6, IL-8, IL-1β, and TNF-α across the experimental groups (Figure 8F). Western blot analysis revealed that, compared with the inflamed control group, glutamine supplementation significantly upregulated the expression of glutamine metabolism-related proteins GLS and SLC1A5, while concurrently downregulating tight junction proteins ZO-1 and Occludin. Notably, BPTES treatment reversed these Gln-induced alterations, restoring tight junction protein levels and suppressing glutamine metabolic protein expression (Figure 8G).

## 4 Discussion

RE, a prevalent and recurrent subtype of GERD, is pathologically defined by esophageal mucosal inflammation and erosion secondary to the retrograde reflux of gastroduodenal contents (Bertin et al., 2025). The traditional acid-centric etiological paradigm, which centers on lower esophageal sphincter dysfunction and excessive gastric acid secretion, has long dominated clinical practice with a primary focus on acid-suppressive therapies (Kikuchi et al., 2021). However, the high incidence of PPI resistance and disease recurrence underscores the inherent limitations of this acid-driven model, a challenge that has been increasingly recognized and validated in recent clinical and translational studies (Musuuza et al., 2021). Accumulating evidence from metagenomic profiling and clinical cohort studies has established that gut microbiota dysbiosis and the intricate crosstalk between gut microbes and host metabolism play pivotal roles in the pathogenesis of various gastrointestinal inflammatory disorders, including inflammatory bowel disease and chronic gastritis (Zhang et al., 2022). Building on this framework, the present study integrated multi-omics analyses (gut microbiome and serum metabolomics) with *in vitro* cellular experiments to identify the gut microbiota-glutamine axis as a key regulatory node in RE pathogenesis. This finding not only provides a novel theoretical basis for understanding RE beyond the conventional acid-centric paradigm but also complements and extends existing research on gut-esophageal axis dysregulation in RE.

A core finding of the present study reveals that gut microbiota dysbiosis is a key feature in the pathogenesis of RE. As a central component maintaining gastrointestinal microecological homeostasis, gut microbiota dysbiosis is not only closely associated with the pathological processes of GERD, contributing to gut-esophageal axis dysfunction, but also serves as a crucial mechanism driving GERD and related esophageal mucosal injury (Guan et al., 2025; Wang et al., 2024). Compositional analysis of the gut microbiota revealed characteristic alterations in RE patients. At the phylum level, the relative abundances of *Bacteroidota* and *Pseudomonadota* were increased, while those of *Bacillota* and *Actinomycetota* were decreased. Notably, the depletion of *Bacillota* and enrichment of *Pseudomonadota* are directly linked to the impaired intestinal barrier function in RE patients. This is attributed to the fact that *Bacillota* is enriched with short-chain fatty acid (SCFA)-producing bacteria, which are key guardians of the intestinal barrier (Czarnowski et al., 2024; Pérez-Reytor et al., 2021). In contrast, *Pseudomonadota*, as a typical pro-inflammatory phylum, can trigger persistent intestinal inflammation and disrupt intestinal mucosal immune homeostasis when overgrown (Prame Kumar et al., 2023; Schult-Hannemann et al., 2025). However, metabolite-microbiota correlation analysis provided more refined insights: within the phylum *Pseudomonadota*, specific genera including *Limnobacter*, *Shewanella*, and *Tropicimonas* exhibited significant negative correlations with Glu-Glu. This indicates that despite the overall increased abundance of *Pseudomonadota*, distinct taxa within this phylum undergo differential regulation—pro-inflammatory taxa expand while potentially beneficial taxa are suppressed. *Shewanella* species are recognized for their metabolic versatility and immunomodulatory properties; the probiotic strain *S. putrefaciens* SpPdp11 has been demonstrated to downregulate pro-inflammatory cytokines while enhancing anti-inflammatory responses and improving intestinal barrier function (Cámara-Ruiz et al., 2020). Similarly, *Acrocarpospora* within the phylum *Actinomycetota* also showed negative correlation with Glu-Glu. This genus, belonging to the phylum *Actinomycetota*, has been demonstrated to produce bioactive metabolites with potent anti-inflammatory activity, including inhibition of NO generation and suppression of pro-inflammatory cytokines (iNOS, TNF-α, IL-6) in macrophages (Wu and Cheng, 2022), further supporting that the depletion of this phylum in reflux esophagitis is associated with metabolic dysregulation rather than merely overall reduced abundance.

At the family and genus levels, the characteristic features of gut microbiota dysbiosis in RE patients further highlight its association with esophageal inflammation: there was a marked enrichment of pro-inflammatory taxa (*Bacteroidaceae*/*Bacteroides*, *Prevotellaceae*/*Prevotella*, *Streptococcaceae*/*Streptococcus*, *Enterobacteriaceae*) and depletion of beneficial taxa (*Bifidobacteriaceae*/*Bifidobacterium*, *Lachnospiraceae*, *Oscillospiraceae*, *Roseburia*). Particularly, *Escherichia coli* and *Klebsiella pneumoniae* were identified as the dominant signature microbes in oesophageal adenocarcinoma (EAC) patients (Zaidi et al., 2022), whereas Faecalibacterium prausnitzii and *Bifidobacterium* adolescentis were significantly depleted (Liu et al., 2024). Mechanistically, *E. coli* and *K. pneumoniae* can activate the intestinal mucosal innate immune pathway by producing LPS, inducing the release of pro-inflammatory cytokines and triggering systemic inflammatory cascades. These inflammatory signals can spread to the esophageal mucosa through the gut-esophageal axis, directly exacerbating esophageal mucosal injury in RE patients. In contrast, *F. prausnitzii* and B. adolescentis exert protective effects through distinct mechanisms: *F. prausnitzii* maintains normal crypt morphology and protects the structural integrity of the colonic mucosa, preserving the epithelium as a physical barrier against bacterial translocation (Lapiere et al., 2020); B. adolescentis enhances intestinal competitiveness through bile salt hydrolase-mediated deconjugation of bile salts, generating unconjugated bile acids with antimicrobial properties against gram-negative pathogens (Jones et al., 2008; Ruiz et al., 2013). The depletion of these beneficial bacteria therefore compromises both physical barrier integrity and colonization resistance, facilitating the translocation of gut microbes and inflammatory mediators to the esophagus—an important contributor to the chronicity of esophageal inflammation in GERD patients. The depletion of these beneficial bacteria directly impairs the “defensive function” of the intestinal barrier, facilitating the translocation of gut microbes and inflammatory factors to the esophagus—an important contributor to the chronicity of esophageal inflammation in GERD patients. Collectively, the characteristic gut microbiota dysbiosis participates deeply in the pathogenesis of RE through the core pathway of “disrupting intestinal barrier-inducing systemic inflammation–targeting esophageal injury,” providing critical evidence for deciphering the pathological regulatory network of RE from the perspective of the gut-esophageal axis.

Gut microbiota dysbiosis in RE extends beyond compositional alterations; the consequent functional perturbations serve as a core driver of RE pathogenesis. By disrupting host metabolic homeostasis and intestinal barrier function, these functional abnormalities are deeply involved in the pathological progression of both RE and GERD. Compared with mere compositional differences, functional remodeling of the gut microbiota more accurately reflects its regulatory role in esophageal inflammation—a feature fully corroborated by KEGG, eggNOG, and CAZy functional annotations in RE patients, which delineates the complete pathogenic pathway by which microbiota transitions from “compositional imbalance” to “functional aberration” to drive RE development.

From the perspective of metabolic regulation, the significant enrichment of carbohydrate and amino acid metabolism pathways in the gut microbiota of RE patients directly mediates host nutrient metabolic disorders, thereby regulating esophageal mucosal inflammatory responses. Metabolic reprogramming of carbohydrates and amino acids by the gut microbiota can alter the composition of immunomodulatory metabolite pools, influencing systemic immune homeostasis (Han et al., 2025)—an imbalance of which is a key inducer of persistent esophageal mucosal inflammation in RE. Notably, the dysregulation of amino acid and purine metabolites observed in RE patients in this study is a direct manifestation of abnormal microbial metabolic function. Such metabolic disorders have been confirmed to be closely associated with the degree of esophageal mucosal injury in GERD patients, suggesting that microbiota-mediated metabolic imbalance may be a common pathological basis for both RE and GERD.

In terms of material transport and environmental adaptation, the downregulation of ABC transporters, carbohydrate transporters, and ATPases in the gut microbiota of RE patients severely impairs microbial nutrient uptake and metabolite exchange between the host and microbiota. This transport dysfunction not only disrupts local intestinal nutrient homeostasis but also breaks the symbiotic balance between the host and microbiota, exacerbating intestinal microecological disorders (Baek et al., 2023). Such imbalances can further amplify esophageal mucosal inflammatory responses through the gut-esophageal axis. In contrast, the upregulation of genes related to bacterial membrane components and redox homeostasis reflects adaptive changes of the microbiota in the inflammatory microenvironment of RE. Although this adaptive remodeling enables microbial survival in the inflammatory milieu, it further enhances their pro-inflammatory phenotypes, forming a vicious cycle of “inflammatory microenvironment–microbial adaptive changes–aggravated inflammation” that accelerates RE progression. Furthermore, characteristic alterations in polysaccharide-degrading enzymes of the gut microbiota in RE patients also contribute to the pathological regulation of RE. As key tools for the microbiota to metabolize dietary polysaccharides and host-derived glycans, dysregulation of these enzymes directly alters the production of core metabolites in the intestine (Liu et al., 2019). These metabolites act as important regulatory mediators of the gut-esophageal axis; their abnormal secretion can affect esophageal mucosal barrier function and inflammatory status through the circulatory system or intestinal mucosal immune pathways. This further confirms that microbial functional remodeling deeply participates in RE pathogenesis by regulating metabolic signals of the gut-esophageal axis.

The gut microbiota-glutamine axis identified by multi-omics integration serves as a critical functional hub for deciphering the role of gut microbiota in RE pathogenesis. Its core value lies in clarifying the specific pathway through which microbiota drive esophageal inflammation by regulating amino acid metabolic reprogramming, greatly expanding the research dimension of the association between gut microbiota and gastrointestinal diseases. Distinct from generalized microbiota-metabolism crosstalk, this axis precisely links RE-characteristic microbiota imbalance to esophageal mucosal inflammatory injury, providing a novel functional target for understanding the gut-esophageal axis regulatory mechanism in RE. This axis acts as a central mediator for transmitting intestinal microecological disorders to esophageal injury in RE patients. The negative correlation between RE-enriched microbes and Glu-Glu, a key intermediate product of glutamine metabolism, confirms that microbiota imbalance can actively regulate host glutamine metabolism. The abnormal activation of the glutamine metabolic pathway in inflammatory HEECs is the direct local manifestation of microbiota regulatory signals in the esophagus. As an essential amino acid regulating intestinal barrier integrity and immune homeostasis, glutamine upregulation in RE initially serves as a compensatory response of the host against esophageal mucosal injury. However, persistent gut microbiota imbalance drives glutamine metabolism toward pathological and sustained upregulation (Zhu et al., 2023), creating a self-amplifying cycle that disrupts immune-metabolic homeostasis and accelerates RE progression. The enrichment of pathogenic bacteria (*Escherichia coli*, *Klebsiella pneumoniae*) and depletion of beneficial symbionts (Faecalibacterium prausnitzii, *Bifidobacterium* adolescentis) converge on a common metabolic endpoint: elevated host glutamine availability. Rather than simply meeting bioenergetic demands, this glutamine surge serves as a metabolic programming signal that favors Th17 differentiation over regulatory T cell induction (Johnson et al., 2018). Simultaneously, the loss of microbial competitors through dysbiosis reduces commensal consumption of luminal glutamine (Tingler et al., 2026), further increasing substrate availability for pathogenic utilization and host inflammatory responses. This dual mechanism—enhanced host production coupled with diminished microbial consumption—establishes a pathological glutamine pool that perpetuates mucosal inflammation.

Specifically, gut microbiota-induced glutamine metabolism activation triggers esophageal epithelial inflammation through two key pathways. First, glutamate, an intermediate metabolite of glutamine, modulates dendritic cell function and maturation status through metabotropic glutamate receptor signaling, thereby influencing naive T cell activation and Th17 lineage commitment (Oner et al., 2021). The gut microbiota-glutamine axis thus converts intestinal microecological disturbance signals into local esophageal inflammatory responses through this immunometabolic reprogramming. Second, α-ketoglutarate (α-KG), another metabolite of glutamine, serves as a critical substrate for prolyl hydroxylase (PHD) activity while its accumulation relative to succinate stabilizes hypoxia-inducible factor-1α (HIF-1α), which in turn activates the mammalian target of rapamycin complex 1 (mTORC1) pathway (Du et al., 2025), inducing metabolic reprogramming of inflammatory cells and forming a positive feedback loop that amplifies esophageal mucosal inflammation and accelerates RE progression.

More importantly, the gut microbiota-glutamine axis can further amplify its pathogenic effects on RE through multi-pathway crosstalk. On one hand, the correlation between Glu-Glu, choline bitartrate (a membrane integrity-related molecule), and 1,3-dimethyluracil (a microbial metabolite) suggests that this axis can indirectly affect the defensive capacity of the esophageal mucosa by regulating intestinal mucosal barrier function and the homeostasis of microbial metabolites. On the other hand, the close association of this axis with glutathione metabolism and arginine biosynthesis pathways further expands the mechanistic dimension of gut microbiota-regulated RE. Persistent pathological upregulation of glutamine impairs glutathione synthesis, reduces the antioxidant capacity of the esophageal mucosa, and renders it more susceptible to inflammatory factor-induced damage. Meanwhile, abnormal nitric oxide release mediated by arginine metabolism disorders—nitric oxide being a key mediator of esophageal mucosal injury in RE—can directly exacerbate esophageal epithelial cell damage and inflammatory responses. Collectively, the gut microbiota-glutamine axis constructs a complete pathogenic chain linking gut microbiota imbalance to esophageal mucosal injury through the “activation of core inflammatory pathways–amplification via multi-pathway crosstalk” mode. This not only deepens the understanding of RE pathogenesis but also provides a novel mechanism-based target for breaking through existing therapeutic bottlenecks.

This study has several limitations, most of which are common in cross-sectional and *in vitro* studies related to gut microbiota and metabolic diseases. First, the sample size was imbalanced between the HC group (*n* = 15) and the RE group (*n* = 35), which may have affected statistical power. Although stringent statistical approaches, including false discovery rate correction for multiple comparisons and effect size prioritization, were implemented to control false-positive risks, this sample size disparity may still have limited the detection sensitivity of low-abundance differential features. Future investigations will validate the current findings by expanding the healthy control cohort or employing a matched design. The cross-sectional design cannot establish a causal relationship between gut microbiota dysbiosis, the gut microbiota-glutamine axis, and RE progression. Longitudinal studies are needed to track dynamic changes during RE occurrence, progression, and treatment to clarify causality, a research direction recommended in previous gut microbiota study guidelines. Second, we acknowledge that the correlation coefficients between Glu-Glu and gut microbial genera were relatively weak. While statistically significant, these weak linear correlations likely reflect complex non-linear or indirect regulatory relationships between gut microbiota and serum metabolites in RE patients, rather than simple linear associations. The biological relevance of these correlations should be interpreted with caution, as they may represent downstream consequences of metabolic dysregulation rather than direct causal relationships. Third, *in vitro* cell experiments cannot fully simulate the complex pathological microenvironment *in vivo*. Future studies using RE animal models are required to further verify the *in vivo* applicability of the complete pathogenic chain, a necessary step for translating *in vitro* findings into clinical applications. Fourth, the impact of confounding factors (diet, lifestyle, PPI use) on gut microbiota and metabolic profiles was not evaluated. Controlling these factors in future studies will enhance the reliability of results, as they are known to affect microbial composition and metabolic pathways. Fifth, the synergistic therapeutic strategy of GLS inhibitors combined with glutamine modulation remains in the preclinical stage, with dose-effect relationships yet to be clarified. Optimization through animal experiments, dose gradient studies, and subsequent clinical trials is essential for clinical translation, representing a standard process in drug development. Meanwhile, the potential diagnostic and therapeutic value of the identified biomarkers and the gut microbiota-glutamine axis requires verification in larger-scale multi-center cohorts. Sixth, the specific molecular mechanisms by which gut microbiota regulate glutamine metabolism were not explored. Future studies should fill this gap through microbial metabolite screening and protein interaction experiments.

In conclusion, through the integration of multi-omics data and *in vitro* cell experiments, this study is the first to identify the gut microbiota-glutamine axis as a key pathogenic mechanism of RE. This finding breaks through the limitations of the traditional acid-centric pathogenic model, deepens the understanding of the pathological mechanism of RE from the perspective of gut-esophageal axis regulation, and provides a novel functional dimension for elucidating the association between intestinal microecology and esophageal inflammation. Furthermore, this study confirms the paradoxical effect of exogenous glutamine and reveals the therapeutic potential of combined intervention with GLS inhibitors, providing direct experimental support for RE treatment strategies targeting metabolic reprogramming—a strategy that has shown promising application prospects in the field of inflammatory diseases. The gut microbiota-glutamine axis possesses dual values as a diagnostic biomarker and a therapeutic target, especially offering a new approach for the precise diagnosis and treatment of RE patients with PPI resistance.

Future studies focusing on *in vivo* mechanism verification of this axis, exploration of the molecular mechanisms by which gut microbiota regulate glutamine metabolism, optimization of GLS inhibitor combination strategies, and their clinical translation will effectively promote the innovation of RE clinical management models, aligning with the development trend of precision medicine for gastrointestinal diseases.

## 5 Conclusion

This study’s core contribution is the identification of the gut microbiota-glutamine axis as a pivotal pathogenic mechanism in RE, which breaks the traditional acid-centric paradigm and provides a novel gut-esophageal regulatory perspective for RE research. Notably, this axis holds dual potential as a diagnostic biomarker and therapeutic target, especially for PPI-resistant RE, while the validated GLS inhibitor-based combination strategy lays a foundation for metabolic reprogramming-targeted RE therapies. Future translational research on this axis is expected to advance RE precision management, aligning with the development trend of gastrointestinal precision medicine.

## Data Availability

The raw metagenomic sequencing dataset generated in this study is publicly available in the NCBI repository (accession number PRJNA1461389; https://www.ncbi.nlm.nih.gov). The metabolomics data are deposited in the MetaboLights repository (study identifier MTBLS14421; https://www.ebi.ac.uk/metabolights).

## Footnotes

1
https://www.mzcloud.org/

2
https://www.genome.jp/kegg/pathway.html

3
https://hmdb.ca/

4
http://www.lipidmaps.org/
