# Supplementary material for: Multi-omics analysis identifies gut microbiota-glutamine axis contributing to the pathogenesis of reflux esophagitis
Source: Front Microbiol. 2026 May 25;17:1805181. doi: 10.3389/fmicb.2026.1805181 (PMC13243242; doi:10.3389/fmicb.2026.1805181)
Supplement: Supplementary file 1 [file Data_Sheet_1.docx]

Supplementary Material

# Supplementary Figures and Tables

## Supplementary Figures


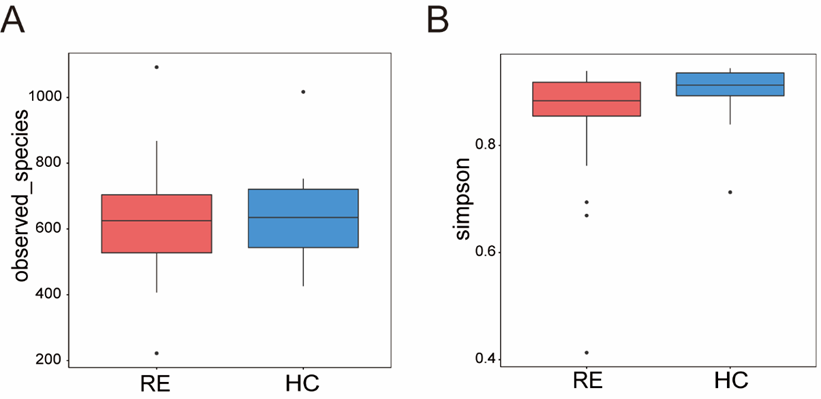


**Supplementary Figure 1.** Analysis of gut microbial diversity between RE patients and HCs based on metagenomic sequencing. **(A)** a-diversity based on the Observed species index between the groups. **(B)** a-diversity based on the Simpson index between the groups.


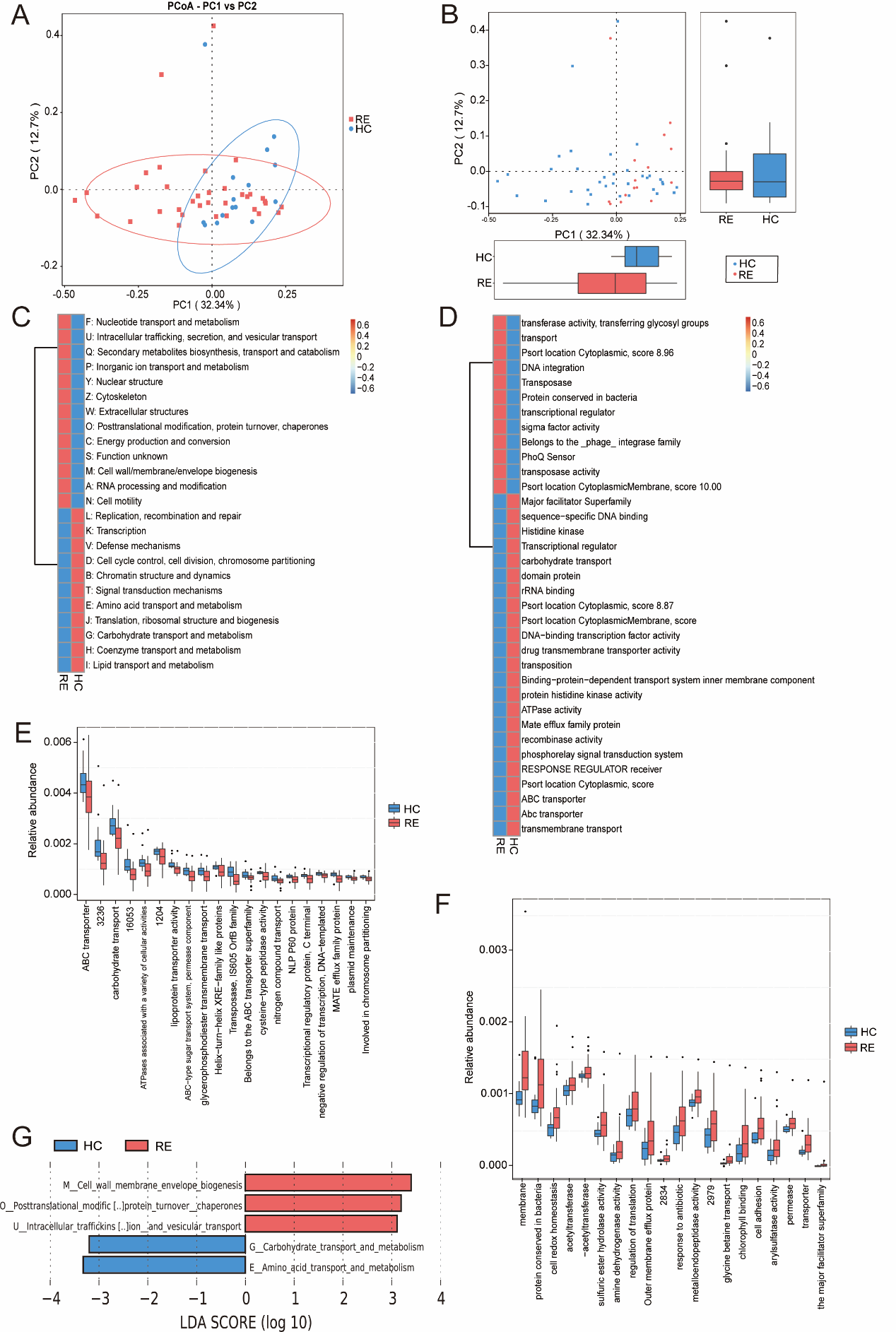


**Supplementary Figure 2.** The eggNOG functional annotation of the gut microbiota genome. **(A)** PCoA on the Bray-Curtis distance; **(B)** NMDS analysis; **(C)**Level 1 clustering heatmap of the KEGG modules; **(D)** Level 2 clustering heatmap of the KEGG modules; **(E, F)** The top 20 with significant differences in abundance at the functional annotation; **(G)** Histogram of the LEfSe analysis based on the level 1 (LDA > 3).


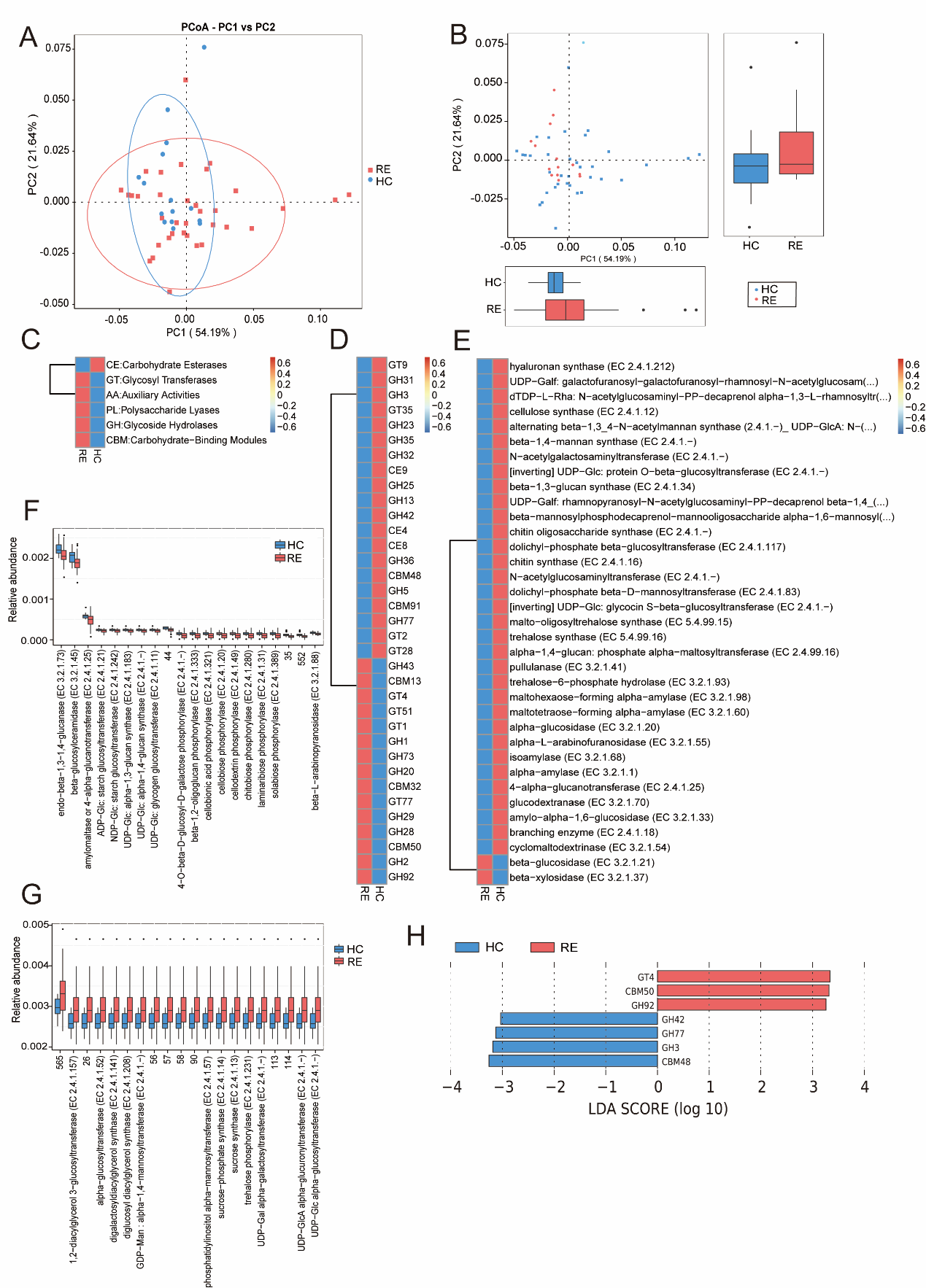


**Supplementary Figure 3.** The CAZy functional annotation of the gut microbiota genome. **(A)** PCoA on the Bray-Curtis distance; **(B)** NMDS analysis; **(C)** Level 1 clustering heatmap of the KEGG modules; **(D)** Level 2 clustering heatmap of the KEGG modules; **(E)** Level 3 clustering heatmap of the KEGG modules; **(F, G)** The top 20 with significant differences in abundance at the functional annotation; **(H)** Histogram of the LEfSe analysis based on the level 2 (LDA>3).

## Supplementary Table

**Table S1. Primer sequences for real-time qPCR in human.**

| Primers | 5`-3` |
| --- | --- |
| GLS | F: TTCAGTCCCGATTTGTGGGG |
|  | R: AAGGAATGCCTTTGATCACCA |
| Myc | F: GGGTAGTGGAAAACCAGCCT |
|  | R: GAGCAGAGAATCCGAGGACG |
| SLC1A5 | F: ATCCATGGGCTCCTGGTACT |
|  | R: CACGCACTTCATCATCAGCG |
| ZO-1 | F: AGCCATTCCCGAAGGAGTTG |
|  | R: GCAAAAGACCAACCGTCAGG |
| Occludin | F: ACTTCAGGCAGCCTCGTTAC |
|  | R: CCTGATCCAGTCCTCCTCCA |
| IL-6 | F: AGACAGCCACTCACCTCTTCAG |
|  | R: TTCTGCCAGTGCCTCTTTGCTG |
| IL-8 | F: GAGAGTGATTGAGAGTGGACCAC |
|  | R: CACAACCCTCTGCACCCAGTTT |
| IL-1β | F: CCACAGACCTTCCAGGAGAATG |
|  | R: GTGCAGTTCAGTGATCGTACAGG |
| TNF-α | F: GAGGCCAAGCCCTGGTATG |
|  | R: CGGGCCGATTGATCTCAGC |
| GAPDH | F: GAAGGTGAAGGTCGGAGT |
|  | R: GAAGATGGTGATGGGATTTC |
